# Hot spring bathing is associated with a lower prevalence of hypertension among Japanese older adults: a cross-sectional study in Beppu

**DOI:** 10.1038/s41598-022-24062-3

**Published:** 2022-11-14

**Authors:** Satoshi Yamasaki, Tomotake Tokunou, Toyoki Maeda, Takahiko Horiuchi

**Affiliations:** 1 Department of Internal Medicine, Kyushu University Beppu Hospital, 4546 Tsurumihara, Tsurumi, Beppu, Oita 874-0838 Japan; 2 Department of Hematology and Clinical Research Institute, National Hospital Organization Kyushu Medical Center, Fukuoka, Japan

**Keywords:** Health care, Hypertension

## Abstract

Hot spring bathing is practiced to help manage various diseases, including hypertension. We investigated the preventive effects on hypertension of hot spring bathing among older adults in a data analysis using responses to a previous questionnaire with the aim to identify a novel approach in the prevention and management of hypertension. Among 10,428 adults aged ≥ 65 years, we assessed the hot spring bathing habits of 4001 individuals with a history of hypertension. We calculated odds ratios (OR) with 95% confidence intervals using a multivariable logistic regression model for history of hypertension. In multivariable logistic regression, age (≥ 85 years: OR, 1.410); history of arrythmia (OR, 1.580), stroke (OR, 1.590), gout (OR, 1.880), diabetes mellitus (OR, 1.470), hyperlipidemia (OR, 1.680), renal disease (OR, 1.520), chronic hepatitis (OR, 0.648); and hot spring bathing at 19:00 or later (OR, 0.850) were independently and significantly associated with hypertension during the lifetime. We found an inverse relationship between habitual nighttime hot spring bathing and a history of hypertension. Prospective randomized controlled trials on nighttime hot spring bathing as a treatment for hypertension are warranted to investigate whether nighttime hot spring bathing can help in preventing hypertension among adults aged ≥ 65 years.

## Introduction

Hypertension is the leading reason for hospital visits and for the long-term use of prescription medications^1–3^. In Japan, ≥ 60% of men aged ≥ 50 years and of women aged ≥ 60 years had hypertension in 2016^4^. In the United States, although the proportion of adults with blood pressure above the target increased from 39 to 53%, the proportion of adults with a recommendation for antihypertensive medication increased from 34 to 36%, according to pooled data from 2011 to 2014. Most patients classified as above require nonpharmacologic intervention as initial therapy^2^. Additionally, owing to multiple stressors encountered within their occupation, groups including military personnel, firefighters, and police officers have an elevated risk for the development of cardiometabolic diseases such as atherosclerosis^5^, heart disease^6^, and sudden cardiac death^7^, which are related to hypertension^8^.

Hot spring bathing has expanded globally from Japan and Asia. Maeda et al. reported preventive effects of the occurrence of hypertension in older women^9^. However, details regarding the relationship between hot spring bathing and hypertension remain unknown.

To address the evidence gap regarding the management of older patients with hypertension, we retrospectively examined the relationship between habitual hot spring bathing and the history of several chronic conditions, including hypertension, in adults aged ≥ 65 years.

## Methods

### Participant selection

We retrospectively reviewed data from an anonymous questionnaire survey (Supplementary Fig. S1) conducted in 2011 with 11,146 responses from individuals aged ≥ 65 years who were living in Beppu city, Japan^9^. In that year, there were 34,465 residents of Beppu city in the age group ≥ 65 years. Questionnaires regarding hot spring bathing habits and disease history were randomly sent to 20,000 residents of Beppu. We analyzed responses from a total of 10,429 survey participants regarding age, sex, disease history, and hot spring bathing habits (Table 1). Of these, we analyzed 10,429 participants who provided valid information regarding age, sex, disease history, and hot spring bathing habits, after we checked all responses in the previously developed and described questionnaire. We also reevaluated all questionnaires, including any descriptions in the free-text response area of the questionnaire. The examined variables were as follows: age; sex; disease history during the lifetime including depression, ischemic heart disease, arrhythmia, hypertension, stroke, gout, asthma, diabetes mellitus, hyperlipidemia, renal disease, chronic hepatitis, collagen disease, and allergy; and hot spring bathing habits, including frequency, duration of immersion, years of habitual bathing, typical time of bathing, and type of hot spring. Informed consent to participate in the study was obtained by providing participants with information on our hospital website. The study was performed in accordance with the institutional guidelines and the principles of the Declaration of Helsinki. The protocol was approved by the institutional review board of Kyushu University Hospital, Japan (No. 24–105).

**Table 1:** Characteristics of survey respondents.

		Total	Hypertension	
Characteristic		n = 10,428	n = 4001	*P*
**Age, n (%)**				
65–69 years old		3121 (29.9)	1072 (26.7)	< 0.001
70–74 years old		2898 (27.7)	1064 (26.6)	
75–79 years old		2514 (24.1)	1019 (25.5)	
80–84 years old		1265 (12.1)	573 (14.3)	
≥ 85 years old		630 ( 6.0)	263 ( 6.8)	
Sex, n (%)	Male	4471 (42.8)	1764 (44.1)	0.048
	Female	5957 (57.1)	2237 (55.9)	
**Disease history, n (%)**				
Cancer		1163 (11.1)	431 (10.8)	0.346
Depression		219 ( 2.1)	88 ( 2.2)	0.625
Ischemic heart disease		724 ( 6.9)	327 ( 8.2)	< 0.001
Arrhythmia		938 ( 8.9)	471 (11.8)	< 0.001
Stroke		354 ( 3.3)	180 ( 4.5)	< 0.001
Gout		416 ( 3.9)	230 ( 5.7)	< 0.001
Asthma		395 ( 3.7)	166 ( 4.1)	0.141
Diabetes mellitus		1427 (13.6)	667 (16.7)	< 0.001
Hyperlipidemia		1154 (11.0)	578 (14.4)	< 0.001
Renal disease		373 ( 3.5)	191 ( 4.8)	< 0.001
Chronic hepatitis		267 ( 2.5)	78 ( 1.9)	0.002
Collagen disease		216 ( 2.0)	86 ( 2.1)	0.710
Allergy		572 ( 5.4)	217 ( 5.4)	0.835
**Hot spring-bathing, n (%)**				
**Frequency**				
< 1/month		2924 (28.0)	1181 (29.5)	0.0013
1/month-1/week		356 ( 3.4)	136 ( 3.4)	
2–3/week		871 ( 8.3)	361 ( 9.0)	
4–5/week		1394 (13.3)	514 (12.8)	
Daily		4884 (46.8)	1809 (45.2)	
**Duration of immersion**				
< 10 min		2645 (25.3)	1108 (27.7)	< 0.001
10–19 min		4304 (41.2)	1613 (40.3)	
20–29 min		2616 (25.0)	973 (24.3)	
≥ 30 min		864 ( 8.2)	307 ( 7.7)	
**Years of habit**				
< 10 years		3021 (28.9)	1225 (30.6)	0.011
10–19 years		1324 (12.6)	486 (12.1)	
20–29 years		1034 ( 9.9)	380 ( 9.5)	
30–39 years		1113 (10.6)	406 (10.1)	
≥ 40 years		3076 (29.4)	1197 (29.9)	
**Time**				
Before 9:00		1272 (12.1)	508 (12.7)	< 0.001
9:00 to 13:00		1145 (10.9)	433 (10.8)	
13:00 to 19:00		4087 (39.1)	1653 (41.3)	
19:00 or later		3925 (37.6)	1407 (35.1)	
**Hot spring type**				
Simple		2193 (21.0)	852 (21.3)	0.864
Chloride		1116 (10.7)	430 (10.7)	0.846
Bicarbonate		329 ( 3.1)	113 ( 2.8)	0.077
Sulfur		87 ( 0.8)	37 ( 0.9)	0.509
Iron		23 ( 0.2)	12 ( 0.3)	0.294
Sulfate		16 ( 0.1)	8 ( 0.2)	0.441
Carbon dioxide		14 ( 0.1)	2	0.095
Acid		1	1	0.384
Aluminum		1	1	0.384

The *p* -values were obtained using the chi-square test.

### Statistical methods

We analyzed the frequencies and descriptive statistics of participants’ variables. Intergroup differences in categorical variables are expressed as number and percentage. The chi-square statistical method was used to test the relationships between categorical variables. Univariable and multivariable logistic regression models were used to determine associations between variables and the prevalence of hypertension. Covariates that were significant at *p* < 0.05 in univariate analysis were included in the multivariate analysis. All tests were two-sided. We calculated 95% confidence intervals (CIs), and *p* < 0.05 was considered statistically significant. Analyses were conducted using EZR (Saitama Medical Center, Saitama, Japan; http://www.jichi.ac.jp/saitama-sct/SaitamaHP.files/statmedEN.html)^10^, which is a graphical user interface for R version 2.13.0 (www.r-project.org), and a modified version of R Commander version 1.6–3 designed to add statistical functions.

### Ethical approval and Consent to Participate

This was a retrospective study with no experimental interventions. The study was approved by the Institutional Review Board of Kyushu University Hospital in Japan. The study was performed in accordance with the institutional guidelines and principles of the Declaration of Helsinki. Informed consent was obtained from all individual participants included in the study.

## Results

Overall, 4001 of 10,428 participants (38.3%) had a history of hypertension. The baseline characteristics of participants with hypertension during the lifetime are presented in Table 1. In a univariable logistic regression model, age ≥ 85 years (odds ratio [OR], 1.460; 95% CI, 1.230–1.740; *p* < 0.001), female sex (OR, 0.923; 95% CI, 0.852–0.999; *p* = 0.048), arrhythmia (OR, 1.610; 95% CI, 1.400–1.850; *p* < 0.001), stroke (OR, 1.620; 95% CI, 1.310–2.010; *p* < 0.001), gout (OR, 1.860; 95% CI, 1.310–2.010; *p* < 0.001), diabetes mellitus (OR, 1.460; 95% CI, 1.300–1.630; *p* < 0.001), hyperlipidemia (OR, 1.650; 95% CI, 1.460–1.870; *p* < 0.001), renal disease (OR, 1.520; 95% CI, 1.230–1.880; *p* < 0.001), chronic hepatitis (OR, 0.656; 95% CI, 0.500–0.859; *p* = 0.002), hot spring bathing frequency (2–3 times/week: OR, 1.200; 95% CI, 1.040–1.390; *p* = 0.013 and daily: OR, 0.868; 95% CI, 0.790–0.954; *p* = 0.003), duration of immersion (10–19 min: OR, 0.832; 95% CI, 0.753–0.918; *p* < 0.001 and 20–29 min: OR, 0.765; 95% CI, 0.652–0.897; *p* < 0.001)), bathing time (13:00 to 19:00: OR, 1.220; 95% CI, 1.110–1.330; *p* < 0.001 and 19:00 or later: OR, 0.840; 95% CI, 0.738–0.957; *p* = 0.008), and years of habitual hot spring bathing (10–19 years: OR, 0.851; 95% CI, 0.745–0.973; *p* = 0.017) were significantly associated with influencing hypertension during the lifetime (Table 2). In a multivariable logistic regression model, age (≥ 85 years: OR, 1.410; 95% CI, 1.170–1.680; *p* < 0.001), history of arrythmia (OR, 1.580; 95% CI, 1.380–1.810; *p* < 0.001), stroke (OR, 1.590; 95% CI, 1.280–1.980; *p* < 0.001), gout (OR, 1.880; 95% CI, 1.530–2.310; *p* < 0.001), diabetes mellitus (OR, 1.470; 95% CI, 1.310–1.650; *p* < 0.001), hyperlipidemia (OR, 1.680; 95% CI, 1.480–1.910; *p* < 0.001), renal disease (OR, 1.520; 95% CI, 1.230–1.880; *p* < 0.001), chronic hepatitis (OR, 0.648; 95% CI, 0.494–0.851; *p* = 0.001); and hot spring bathing at 19:00 or later (OR, 0.850; 95% CI, 0.768–0.940; *p* = 0.001) were independently and significantly associated with the prevalence of hypertension during the lifetime (Table 2). These findings support the hypothesis that hypertension can be influenced by habitual nighttime hot spring bathing.

**Table 2:** Univariable and multivariable analysis of variables influencing history of hypertension.

	Univariable			Multivariable		
Variable	OR	95% CI	*p*	OR	95% CI	*p*
Age 65–69 years old	1.000	Reference				
70–74 years old	1.110	0.998–1.230	0.055			
75–79 years old	0.891	0.747–1.060	0.202			
80–84 years old	1.080	0.893–1.310	0.418			
≥ 85 years old	1.460	1.230–1.740	< 0.001	1.410	1.170–1.680	< 0.001
Sex Male	1.000	Reference				
Female	0.923	0.852–0.999	0.048	1.030	0.949–1.120	0.454
**Disease history**						
Cancer	0.939	0.828–1.070	0.330			
Depression	0.970	0.733–1.280	0.830			
Ischemic heart disease	1.150	0.985–1.350	0.076			
Arrhythmia	1.610	1.400–1.850	< 0.001	1.580	1.380–1.810	< 0.001
Stroke	1.620	1.310–2.010	< 0.001	1.590	1.280–1.980	< 0.001
Gout	1.860	1.520–2.280	< 0.001	1.880	1.530–2.310	< 0.001
Asthma	1.130	0.914–1.390	0.265			
Diabetes mellitus	1.460	1.300–1.630	< 0.001	1.470	1.310–1.650	< 0.001
Hyperlipidemia	1.650	1.460–1.870	< 0.001	1.680	1.480–1.910	< 0.001
Renal disease	1.520	1.230–1.880	< 0.001	1.520	1.230–1.880	< 0.001
Chronic hepatitis	0.656	0.500–0.859	0.002	0.648	0.494–0.851	0.001
Collagen disease	1.030	0.774–1.360	0.861			
Allergy	0.942	0.789–1.130	0.509			
**Hot spring-bathing frequency**						
< 1/month	1.000	Reference				
1/month-1/week	0.912	0.728–1.140	0.427			
2–3/week	1.200	1.040–1.390	0.013	1.050	0.877–1.250	0.619
4–5/week	0.994	0.879–1.120	0.924			
Daily	0.868	0.790–0.954	0.003	1.050	0.930–1.180	0.455
**Duration of immersion**						
< 10 min	1.000	Reference				
10–19 min	0.832	0.753–0.918	< 0.001	0.952	0.825–1.100	0.504
20–29 min	0.765	0.652–0.897	< 0.001	0.950	0.807–1.120	0.535
≥ 30 min	0.931	0.793–1.090	0.380			
**Time**						
Before 9:00	1.000	Reference				
9:00 to 13:00	0.915	0.776–1.080	0.286			
13:00 to 19:00	1.220	1.110–1.330	< 0.001	0.973	0.853–1.110	0.680
19:00 or later	0.840	0.738–0.957	0.008	0.850	0.768–0.940	0.001
**Years of habit**						
< 10 years	1.000	Reference				
10–19 years	0.851	0.745–0.973	0.017	0.921	0.815–1.040	0.183
20–29 years	1.050	0.869–1.270	0.622			
30–39 years	1.040	0.861–1.250	0.706			
≥ 40 years	1.150	0.982–1.350	0.082			
**Hot spring type**						
Simple	0.982	0.889–1.080	0.714			
Chloride	0.977	0.858–1.110	0.722			
Bicarbonate	0.805	0.638–1.010	0.065			
Sulfur	1.150	0.752–1.760	0.515			
Iron	1.590	0.712–3.540	0.258			
Sulfate	1.590	0.595–4.240	0.356			
Carbon dioxide	0.265	0.059–1.180	0.081			

*OR* odds ratio; *CI* confidence interval.

^†^Univariable or multivariable competing event statistics analyzed using a logistic regression model were applied to a positive history of hypertension. Covariates significant at *p* < 0.05 in univariate analysis were included in the multivariate analysis.

## Discussion

Traditional thermal therapy and hot spring bathing have proven useful for various diseases, including hypertension^11^. We investigated the preventive effects of long-term hot spring bathing in adults aged ≥ 65 years. We found that age ≥ 85 years; history of arrythmia, stroke, gout, diabetes mellitus, hyperlipidemia, and renal disease were independently and significantly associated with a higher risk of developing hypertension during the lifetime. We found that a history of chronic hepatitis and hot spring bathing time were independently and significantly protective against hypertension development during the lifetime. These results support our hypothesis that habitual nighttime hot spring bathing is protective against hypertension development.

The implications of our data can be extrapolated regarding the prevalence of hypertension according to questionnaire survey responses from adults aged ≥ 65 years. We found that nighttime hot spring bathing, which can improve sleep disorders, might be inversely associated with a history of hypertension in adults aged ≥ 65 years. In a large-scale study among an older population, Tai et al. reported that nighttime hot spring bathing was significantly associated with shorter sleep onset latency, if bathing is scheduled 1–3 h before bedtime, and higher distal–proximal skin temperature gradient if bathing takes place 30 min before bedtime^12^. Sawatari et al. suggested that leg thermal therapy could improve subjective and objective sleep quality in patients with chronic heart failure^13^. The COVID-19 pandemic has placed all people at risk for developing psychiatric and mental health disorders, including sleep disturbances^14^. It is therefore possible that nighttime hot spring bathing may improve sleep, which may result in improved hypertension control^15^.

According to our data, age ≥ 85 years was significantly associated with the prevalence of hypertension. Many risk factors are associated with hypertension development, such as age, obesity, family history, high-sodium diet, and physical inactivity^16,17^. Disease history, stroke^18^, and renal disease^19,20^ are associated with hypertension. Given that health care spending on hypertension exceeded USD 70 billion in the United States between 1996 and 2016^21^, clinicians and researchers have great interest in proactive and preventive interventions versus reactive approaches for hypertension. In this study, we demonstrated that an alternative option for potentially improving hypertension control in adults aged ≥ 65 years is habitual nighttime hot spring bathing.

Stress has two components: an acute phase and a chronic phase^22^. Rozanski et al. verified a direct association between cardiovascular disease and chronic stress, which is known to modulate vascular endothelial cell function and platelet aggregation^23^. Dual stressors include psychological and physiological stressors and are known to elicit activation of the hypothalamic and sympathoadrenal axes, with a subsequent greater release of stress markers such as cortisol, epinephrine, and norepinephrine, in comparison with a single stressor^24,25^. Increased levels of cortisol and oxidative stress in the body can upregulate several proinflammatory pathways, which can result in the development of several cardiovascular diseases including hypertension^26^. Endocrine responses to sauna bathing show that some markers of stress, such as cortisol, β-endorphins, and adrenocorticotropic hormone, respond to acute heat exposure in a highly variable manner^27^. Different results regarding the hormone response are likely owing to differences in study methods and consideration of factors such as therapy duration, time, and frequency, which were considered in our study. Therefore, understanding the cardiovascular responses will provide a more comprehensive picture of the physiological responses to hot spring bathing. Blood pressure after a sauna bath appears decreased compared with that before a sauna bath^28^. Although brief exposure to sauna baths can result in benefits for < 1 h, including reduced blood pressure and improved arterial stiffness, sauna bath exposure for ≥ 3 weeks and repeated frequency can upregulate enzymes and pathways, which results in greater stress tolerance, an enhanced cellular environment, and improved health^29^. Owing to a lack of physical activity and healthy nutrition and with time pressure among individuals aged ≥ 65 years, practical interventions that can prevent or improve hypertension, such as habitual hot spring bathing, warrant additional attention.

Our study has some limitations that should be acknowledged. First, some selection bias is expected with use of questionnaire surveys. In this study, bias is present owing to differences in data selection and other factors, including a lack of data regarding hypertensive patients who engaged in hot spring bathing for the treatment of hypertension; participant incomes, which might be correlated with some vascular diseases or the frequency of hot spring bathing; and regarding participants’ lifestyle, such as diet, physical activity, and sleep. Second, important data regarding the prevalence of hypertension are likely missing because the information was collected using self-report questionnaires. To minimize bias, we limited the inclusion criteria to age, sex, disease history, and hot spring bathing habits. Third, we have no data about the treatment and outcomes of hypertension; further studies are needed to assess the details of treatment and outcomes in patients with hypertension. Fourth, a history of hypertension may have been overlooked or underreported in some study participants because of the self-report nature of the questionnaire; moreover, diagnoses of disease history, including hypertension, were not confirmed by a physician. Finally, the purpose of this study was to help clarify the relationship between the prevention of hypertension and habitual hot spring bathing. However, it was difficult to interpret the obtained evidence, such as the interactions among duration of immersion, frequency, time, and years of habitual hot spring bathing, because we did not evaluate the quality of the questionnaire data.

## Conclusions

In this study, we found that habitual nighttime hot spring bathing was significantly associated with a lower prevalence of hypertension in older adults. It is important to prioritize clinical trials regarding the prevalence of hypertension, including identifying effective approaches to the monitoring and management of arrhythmia, stroke, gout, diabetes mellitus, hyperlipidemia, renal disease, and chronic hepatitis. Randomized controlled trials on habitual nighttime hot spring bathing as a treatment for hypertension are warranted.

## Supplementary Information

Supplementary Information.

## Data Availability

We used data obtained from a questionnaire performed in 2011 in Beppu, Japan. The datasets generated and analyzed during the current study are not publicly available owing to privacy and confidentiality restrictions pertaining to personal health information. However, the dataset creation plan is available from the corresponding author on reasonable request.
