# Author Correction: Hot spring bathing is associated with a lower prevalence of hypertension among Japanese older adults: a cross-sectional study in Beppu

**DOI:** 10.1038/s41598-022-26365-x

**Published:** 2022-12-22

**Authors:** Satoshi Yamasaki, Tomotake Tokunou, Toyoki Maeda, Takahiko Horiuchi

**Affiliations:** 1 Department of Internal Medicine, Kyushu University Beppu Hospital, 4546 Tsurumihara, Tsurumi, Beppu, Oita 874-0838 Japan; 2 Department of Hematology and Clinical Research Institute, National Hospital Organization Kyushu Medical Center, Fukuoka, Japan

Correction to: *Scientific Reports* 10.1038/s41598-022-24062-3, published online 14 November 2022

The original version of this Article contained an error in the Abstract.

“We found an inverse relationship between habitual nighttime hot spring bathing and a history of depression.”

now reads:

“We found an inverse relationship between habitual nighttime hot spring bathing and a history of hypertension.”

The original Article has been corrected.

