# Supplementary material for: Hot spring bathing is associated with a lower prevalence of hypertension among Japanese older adults: a cross-sectional study in Beppu
Source: Sci Rep. 2022 Nov 14;12:19462. doi: 10.1038/s41598-022-24062-3 (PMC9661464; doi:10.1038/s41598-022-24062-3)
Supplement: Supplementary file 1 — Supplementary Information. [file 41598_2022_24062_MOESM1_ESM.docx]

Supplementary Materials
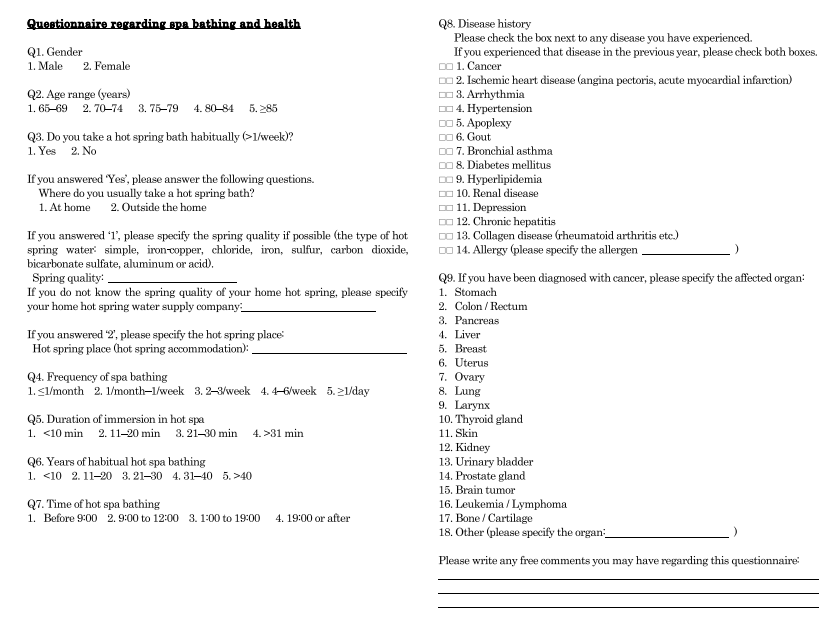
 **Supplementary Figure 1.**

The English version of the Japanese questionnaire used in this study.
